# Zn (II)-porphyrin-based photochemically green synthesis of novel ZnTPP/Cu nanocomposites with antibacterial activities and cytotoxic features against breast cancer cells

**DOI:** 10.1038/s41598-022-21446-3

**Published:** 2022-10-12

**Authors:** Sajedeh Tehrani Nejad, Rahmatollah Rahimi, Mahboubeh Rabbani, Sadegh Rostamnia

**Affiliations:** 1 Inorganic Group, Department of Chemistry, Iran University of Science and Technology (IUST), PO Box 16846-13114, Tehran, Iran; 2 Organic and Nano Group (ONG), Department of Chemistry, Iran University of Science and Technology (IUST), PO Box 16846-13114, Tehran, Iran

**Keywords:** Chemistry, Materials science

## Abstract

This study focuses on synthesizing novel nanocomposites, zinc(II)tetrakis(4-phenyl)porphyrin/Cu nanoparticles (ZnTPP/Cu-NPs),with antibacterial activity, fabricated through a single-step green procedure. In this regard, the self-assembly of ZnTPP was carried out through an acid–base neutralization method to prepare ZnTPP nanoparticles. Then, the copper nanoparticles (Cu-NPs) were grown on ZnTPP nanoparticles through a visible-light irradiated photochemical procedure in the absence and presence of polyacrylic acid (PAA) as a modulator. The effect of PAA on the morphological properties of the prepared nanocomposites was evaluated. Eventually, the antibacterial activity of nanocomposites with different morphologies was investigated. In this way, the average zone of inhibition growth of diameter, minimum inhibitory concentration, and minimum bactericidal concentration values was determined. Besides, the cytotoxicity of the nanocomposites was evaluated by (3-(4,5-dimethylthiazol-2-yl)-2,5-diphenyltetrazolium bromide assay MCF-7and (HEK-293) cell lines. The specific features of the synthesized nanocomposites identified them as antibacterial compounds which have therapeutic effects on breast cancer.

## Introduction

Porphyrins, as biocompatible dispersive macromolecules, due to their specific properties such as having π-conjugated structures and light-sensitive behavior, are known as critical constituents in natural macromolecules such as hemoglobin and chlorophyll^1^. Furthermore, the self-assembly ability of porphyrin-based materials and well-defined geometric structures has increased their applications and capabilities. Among porphyrin-based materials, their metal-based complexes, metalloporphyrins, are of particular importance as recently, their optical characteristics have attracted many researchers' attention. On the other hand, the fact that metalloporphyrins and porphyrins' capability is enhanced in nanoparticle form^2^, has encouraged researchers to control the morphology and size of these nanoparticles because each morphology owns the potential to exhibit different properties^3–6^. Supramolecular interactions such as metal–ligand coordination and π–π stacking assist in preparing porphyrins in the form of nanoparticles. In a study by Bai et al., platinum nanoparticles (Pt-NPs) were synthesized using porphyrin as a template which was removed at the end^7,8^. In another study, Zhang et al. also synthesized copper nanoparticles (Cu-NPs) using the solution of porphyrin and then eliminated the porphyrin from the reaction medium^9^.

In addition to the optical activity, porphyrins can reveal antibacterial features and affect the lipid membrane of gram-negative bacteria through their hydrophobic part. As mentioned, metalloporphyrins, especially hydrophobic species, exhibit antibacterial behavior against gram-positive and gram-negative bacteria. This antibacterial interaction can be conducted by various mechanisms, including oxidase, peroxidase reactions, division, damage to the bacterial membranes, exposure to light, and stimulating and sensitizing bacteria to ROS^10,11^. Chemical modification of porphyrin macromolecules and their nanoparticles is one of the most effective procedures to investigate their antibacterial properties. Besides, these porphyrin compounds usually possess low toxicity against normal cells, but the cytotoxic effect on cancer cells, making them anticancer and antibacterial agents in traceable drugs^12,13^. People with cancer are increasingly susceptible to multi-drug resistant bacteria strains^14–16^. Co-occurrence of chronic infections and cancer is the leading cause of death in many patients. In other words, chronic infections lead to the instability of the immune system, increasing the risk of cancer.

Moreover, the risk of infection and antibiotic resistance in cancer patients can suppress the immune system in the body^13,17,18^ as there is consistently a mutual link between infection and cancer. Hereupon, it is crucial to find and develop compounds compatible with the biological structure of the body with antibacterial activity and cytotoxicity against cancer cells^19,20^. To date, copper-based materials have been extensively used as sterilizers, disinfectants, antibacterial agents, as well as decolorizing photocatalysts^21–24^. Researchers have subscribed that the antibacterial mechanism of Cu depends on the particles' form and size^25^. Cu-based nanoparticles are capable of killing the bacteria by releasing metal ions and exposing the membrane of protein and peptide macromolecules within the cell^26^. Moreover, in case the nanoparticle form of these compounds is stable in suspension, they can be well-suited for drug delivery. Among nanometals, copper (Cu) is a promising candidate for the development of a novel generation of preparations. Interestingly, copper is a trace element and a nontoxic heavy metal to many living cells^27^. On the one hand, copper participates in many important metabolic processes. On the other hand, it observes significant bacteriostatic and bactericidal activity due to cell membrane, nucleic acid, and protein damage^28^.

In continuation of our previous reports on colloidal material in catalysis and biological fields^29–35^, in this study, the visible light illuminated synthesis of colloidal ZnTPP /Cu-NPs nanocomposite was carried out. The synthesized ZnTPP /Cu-NPs nanocomposite from the colloidal stability point of view was investigated by the light scattering method based on the Tyndall effect to determine colloidal particles' stability over time in suspension. From the synthetic point of view, ZnTPP-NPs were first prepared through the injection of acidified porphyrin solution into the surfactant alkaline solution, and their self-assembly was carried out during the acid–base neutralization method which its morphology was also investigated and controlled. To achieve the ZnTPP /Cu-NPs nanocomposite, the visible light illumination synthesis method was performed which on that the ZnTPP-NPs act as a photochemical promoter, ascorbic acid (AA) as an electron donor, and the polyacrylic acid (PAA) is an influencing agent on the morphology with CuCl_2_ solution under halogen lamp irradiation source. Furthermore, the effect of PAA on morphological properties of prepared samples was evaluated in the presence and absence of it. Then, the antibacterial performance of synthesized ZnTPP-NPs and ZnTPP /Cu-NPs was investigated against different strains of gram-positive *Staphylococcus aureus* (*S. aureus*) and gram-negative *Escherichia coli* (*E. coli*) bacteria qualitatively and quantitatively under the same conditions. The cytotoxicity of synthesized compounds was also investigated against HEK-293 and MCF-7 cell lines.

## Experimental

### Materials and methods

Each of chemicals used in this work were of analytical grade. Copper chloride dihydrate (CuCl_2._ 2H_2_O), sodium hydroxide (NaOH), hydrochloric acid (HCl 37%), methylene blue (MB, C_16_H_18_ClN_3_S), ethanol 96%, pyrrole (C_4_H_4_NCH_3_), and propionic acid (CH_3_CH_2_CO_2_) were prepared from Merck Company. Zinc acetate dihydrate (Zn (CH_3_CO_2_)_2_. 2H_2_O) and dimethylformamide (DMF, HCON(CH_3_)_2_) for porphyrin metallation were also prepared from Merck Company. L-ascorbic acid (AA, HC_6_H_7_O_6_) and poly(acrylic acid) (PAA, (CH_2_-CHCO_2_H)_n_), cetyltrimethylammonium bromide (CTAB, C_19_H_42_BrN), were prepared from Sigma-Aldrich Company. Deionized water was utilized to prepare solutions. All of the materials were used with no further purification except pyrrole, which was first distilled and used to synthesize porphyrin.

X-ray diffraction (XRD) analysis was performed on a D Jeoljdx-8030 X-ray powder diffractometer with Cu Kα (l = 0.154 nm) radiation (40 kV, 30 mA). Dynamic light scattering (DLS) analysis was utilized to measure the size of nanoparticles (DLS SZ 100 z) and X-ray photoelectron spectroscopy (XPS) was carried out using a dual anode (Mg and Al Kα) achromatic X-ray source to determine the exitance of the structural elements in nanocomposites structure. To estimate the average size of particles and morphological investigation of the composites and also map scanning and EDS Semi-quantitative elemental analysis, the MIRA3 TESCAN -XMU Field emission electron microscope (FE-SEM) was used. Nanoparticles were determined by using transmission electron microscope (model: EM 208S) AT 100 kV. Incandescent Halogen lamp (DONAR DN-30059, 82 V, 360 W) for photocatalytic synthesis, and additionally pointer laser lamp for Tyndall effect investigation were utilized. The Fourier transform infrared (FT-IR) analyses were carried out on a Shimadzu FTIR-8400S spectrophotometer using a KBr pellet for sample preparation. Surface areas and pore size distribution were determined using Brunauer–Emmett–Teller (BET) multilayer nitrogen adsorption method in a conventional volumetric technique by ASAP 2020 micromeritics instrument. For indicating the porphyrin’s structure of composites, using a double-beam UV–visible spectrometer (Shimadzu UV-1700) at room temperature in the range of 400–800 nm. 0.5 McFarland turbidity standard to Nutrient Broth media was applied for the antibacterial test.

### Preparation of zinc meso-tetraphenyl porphyrin (ZnTPP)

The tetraphenyl porphyrin (H_2_TPP) was prepared through the procedure reported by A.D Adler et al.^36^, first of all, the pyrrole was distilled. Then, 9 mmol of distilled pyrrole and 9 mmol benzaldehyde were refluxed in 170 mL of propionic acid for 4 h. The product was purified by a chromatographic column. Thereafter for preparing ZnTPP, 1 mmol of prepared H_2_TPP and 2 mmol of Zn(Ac)_2_ were refluxed in 70 mL DMF (155 °C) for 6 h.

### Preparation of ZnTPP NPs

ZnTPP nanoparticles were synthesized through the acid–base neutralization self-assembly method. In this way, 10 mL solution of ZnTPP (0.1 M) was dispersed in HCl solution (0.2 M), then was injected into 19 mL of non-stop stirred aqueous solution of cetrimonium bromide-CTAB (0.01 M) and NaOH (0.008 M) at STP conditions. The reaction mixture was stirred for 40 min, then centrifuged at 10,000 rpm, washed with deionized water, and dried in the air.

### Preparation of ZnTPP/Cu-NPs

The ZnTPP/Cu-NPs nanocomposite was synthesized (under glovebox) via two procedures:

i. Initially, 0.5 mL of CuCl_2._ 2 H_2_O (20 mM), 0.5 mL of ascorbic acid (0.1 M), and 10 mL of ZnTPP nanostructures (0.1 g/L), all in the form of a dispersion, introduced to a glass vial. Next, the prepared mixture was subjected to a 360-Watt, 82 Volt halogen light lamp and stirred for 10 min. The obtained product was centrifuged at 7000 rpm for 15 min and then washed with deionized water to remove the surfactant and excess salt. Finally, the synthesized ZnTPP/Cu-NPs were dried. The synthesis of ZnTPP/Cu-NPs was done in a glove box under nitrogen.
ii. In the second procedure, the solutions including 0.5 mL of CuCl_2._ 2 H_2_O (20 mM), 0.5 mL of ascorbic acid (0.1 M), 10 mL of ZnTPP nanostructures (0.1 g/L) and 1 mL of PAA (1.25 mM) were added to a glass vial. Then, the reaction mixture underwent a 360-Watt, 82 Volt halogen light lamp and stirred for 10 min. Eventually, the reaction mixture was centrifuged at 7000 rpm for 15 min, and the obtained product was rinsed with deionized water. Then, it was dried and the obtained powder. Other samples were also synthesized using various amounts of materials which have been outlined in Table 1.

**Table 1:** Synthesized samples in various conditions.

Name	CuCl_2_· 2 H_2_O	Ascorbic acid	PAA
ZnTPP/Cu-NPs (1)	0.5 mL 20 mM	0.5 mL 0.1 M	–
ZnTPP/Cu-NPs-PAA (1)	0.5 mL 20 mM	0.5 mL 0.1 M	0.5 mL 1.25 mM
ZnTPP/Cu-NPs (2)	0.5 mL 50 mM	2 mL 0.1 M	–
ZnTPP/Cu-NPs-PAA (2)	0.5 mL 50 mM	2 mL 0.1 M	0.5 mL 1.25 mM
ZnTPP/Cu-NPs (3)	0.5 mL 100 mM	5 mL 0.1 M	–
ZnTPP/Cu-NPs-PAA (3)	0.5 mL 100 mM	5 mL 0.1 M	0.5 mL 1.25 mM

### Antibacterial activity appraisement

#### Microorganism preparation

To conduct the antimicrobial tests, the strains of gram-negative and gram-positive bacteria were utilized. In this regard, *E. coli* (ATCC 25922) and (PTCC 1399) strains and *S. aureus* (ATCC 25923) and (PTCC 1112) strains were purchased from the microorganism bank of the Scientific Industrial Research Organization of Iran.

#### Well diffusion method

Antibacterial activities of samples were assessed qualitatively by agar well diffusion method protocol in Mueller–Hinton agar medium. To explain, a suspension (0.5 × 10^8^ CFU/mL) containing the bacteria strains, *E. coli* or *S. aureus*, was spread on the Mueller–Hinton agar. Then, wells with 5 mm volume were punched into the nutrient agar plates, and 1 mL (10 mg) of the suspension of each sample was poured onto each well. Afterward, they were incubated at 37 °C for 24 h. The tests were repeated three times for each sample, and the average zones of inhibition were obtained.

#### MIC determination

The standard broth dilution method (CLSI M07-A8) was used as a key method to determine the minimum bacterial growth inhibitory concentration (MIC) of ZnTPP-NPs and ZnTPP /Cu-NPs. Indeed, the visible growth of microorganisms in the agar broth is checked in this procedure. For this purpose, serial dilution of nanocomposites with 0.0390 to 5 mg/mL concentrations was prepared, and their interaction with a specific value of bacterial (10^8^ CFU/mL, 0.5 McFarland's standard) was studied to determine the MIC in BHI broth. The control contained only inoculated broth and was incubated for 24 h at 37 °C.

#### MBC determination

The MBC for antibacterial agents is the lowest concentration needed for killing 99% of the bacterial population. After achieving MIC, a portion of each microplate that lacks visible bacterial growth is seeded on BHI agar plates and incubated for 24 h at 37 °C. The value of MBC is obtained by observing the bacteria present in the incubated agar plates during incubation.

### Cytotoxicity assay

Human embrunic kidney HEK-293 cell linesand MCF-7 cells were bought from the national cell bank of Iran (Pasture Institute, Tehran, Iran).

#### Cell viability assay

MTT test was performed to measure the toxicity of ZnTPP/Cu-NPs (3) and ZnTPP/Cu-NPs-PAA (3) nanocomposites on HEK 293 and MCF-7 cells respectively. 1 × 104 cells were seeded to each well of a 96‐well plate for 24 h. Different concentrations (0, 3.9, 7.8, 15.625, 31.25, 62.5 μg/mL) of PE38, ZnTPP/Cu-NPs (3) and ZnTPP/Cu-NPs-PAA (3) were added to the plate (eight replicates). The plate incubation for 48 h was done. Then, 100 μL of MTT solution (at a final concentration of 0.05 mg/well) was added. Then, 100 μL/well of dimethyl sulfoxide was utilized to solubilize the formed formazan crystals in cells. The absorption was read at 570 nm using a microplate reader.

### Photodegradation test

The photocatalytic degradation of methylene blue (10 mL, 5 ppm) was investigated as an additional test using 2 mg nanocomposite for six hours. This test was carried out using a double beam UV spectrophotometer to evaluate the pre-catalytic properties of composites in the 400–800 nm wavelength range (Fig. S1). The hydroxylation intermediates formed from the environmental water and O_2_ during the oxidation process, is the basis of photodegradation mechanism^37,38^.

### Statistical analysis

Statistical analysis was done using package SPSS v16.0 software. Moreover, Microsoft Excel was utilized for data descriptive analysis.

## Results and discussion

**Figure 1:**
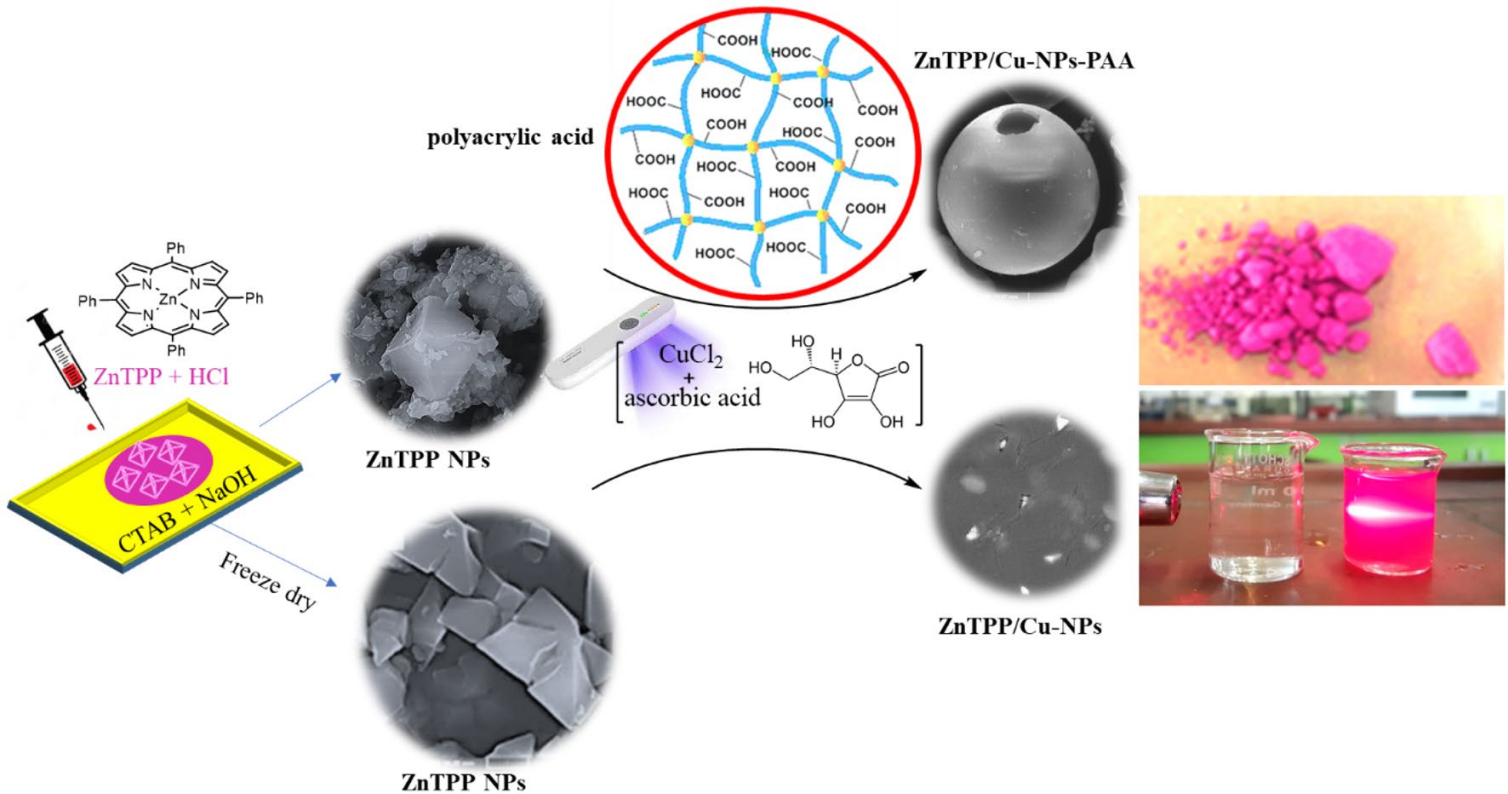
The schematic illustration of ZnTPP, ZnTPP-NPs, ZnTPP/Cu-NPs and ZnTPP/Cu-NPs-PAA preparation.

As mentioned, ZnTPP NPs were synthesized based on the Adler method^36^ followed by self-assembling during the acid–base neutralization approach. ZnTPP/Cu-NPs were then prepared in a single-step visible light illumination process using CuCl_2__._ 2H_2_O, ascorbic acid (AA), and halogen light as the source of irradiation. In addition, during this photochemical reaction, the effect of adding PAA (polyacrylic acid) as a modulator of morphology for ZnTPP/Cu-NPs and ZnTPP/Cu-NPs-PAA nanostructures was also investigated (Fig. 1).

**Figure 2:**
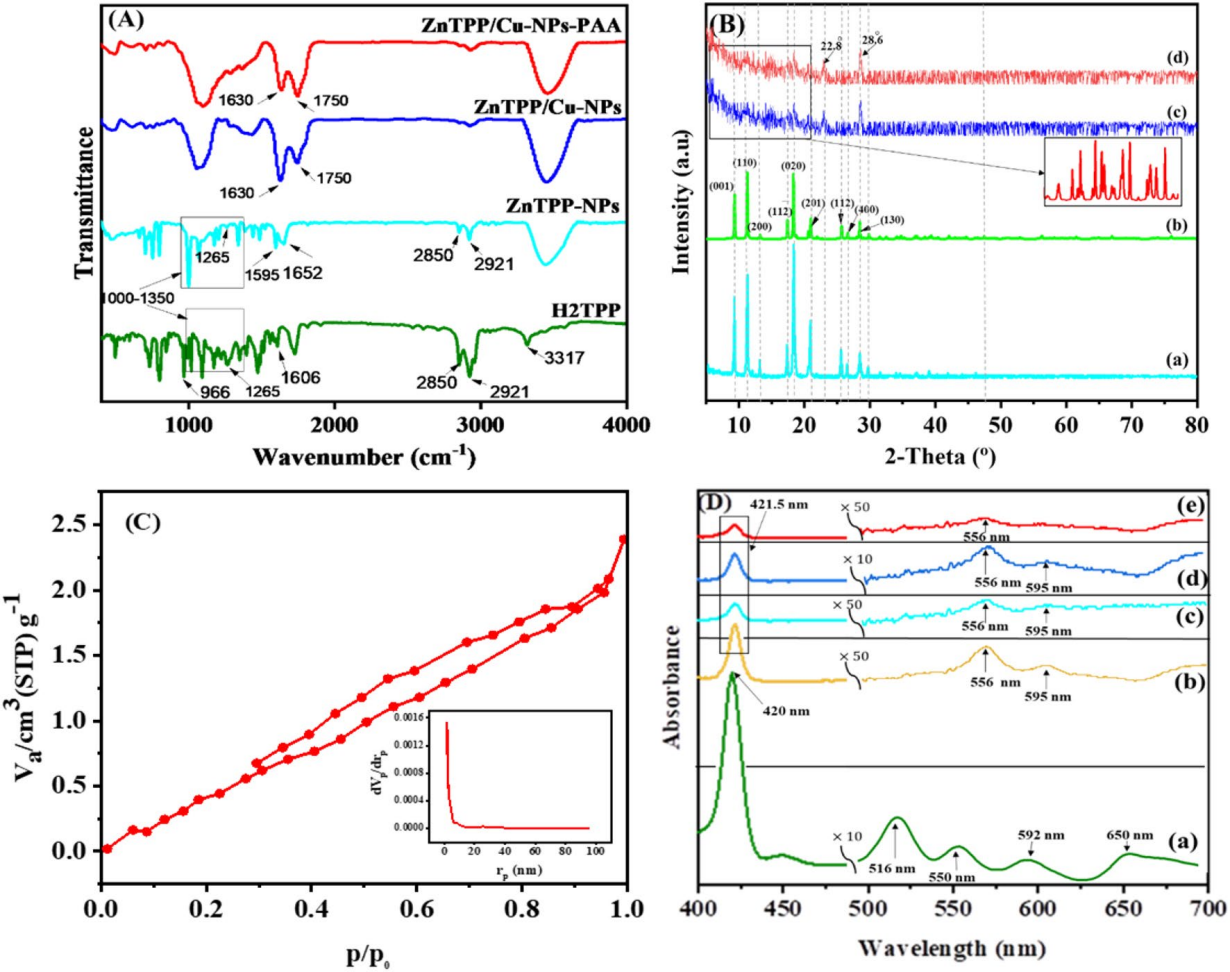
FT-IR, PXRD, BET-BJH, and UV–Vis of prepared porphyrin-based materials.

Figure 2A shows the FT-IR spectra of H_2_TPP, ZnTPP-NPs, ZnTPP/Cu-NPs, and ZnTPP/Cu-NPs-PAA indicating signals at 2850 and 2921 cm^−1^ assigned to the vibrational stretching mode of aliphatic C–H, and signals at 1265 and 1000–1350 cm^−1^ allocated to the vibrational mode of C–N. All these signals are standard in the spectra of both H_2_TPP and ZnTPP-NPs compounds, characterizing the presence of tetraphenyl porphyrin (TPP).On the other hand, signals at 3317and 966 cm^−1^ are ascribed to the stretching and bending vibration of N–H within the pyrrole ring, respectively, and the absence of these two peaks in the spectra of ZnTPP-NPs indicates the successful formation of this complex^39^. Moreover, regarding the spectrum of H_2_TPP, the signal at 1606 cm^−1^, which is attributed to the C = N stretching vibration of pyrrole, splits into two peaks at 1595 and 1652 cm^−1^ indicating the formation of Zn-N coordination in the spectra of the prepared ZnTPP-NPs which have shifted to 1630 and 1750 cm^−1^ in ZnTPP/Cu-NPs and ZnTPP/Cu-NPs-PAA composites^40,41^.

As shown in Fig. 2Ba the XRD pattern of self-assembled ZnTPP (ZnTPP-NPs) reveals diffraction peaks at (001), (110), (200), (112), (020), (201), (400), and (130) corresponding to the simulated pattern from published crystal structure data (CCDC, Ref. code ZNTPOR03), octahedrons (Fig. 2Bb)^42^. Concerning the X-ray diffraction pattern of ZnTPP/Cu-NPs and ZnTPP/Cu-NPs-PAA nanocomposites represents the alteration of the crystalline structure and morphology of ZnTPP-NPs, likely after the formation and modulating of the nanocomposites (Fig. 2Bc, Bd)^43^. The peaks also appeared at 2θ = 22.8 and 28.6° in the XRD pattern of ZnTPP/Cu-NPs and ZnTPP/Cu-NPs-PAA corresponding to the ZnTPP crystalline surfaces (Fig. 2Bc, Bd)^44^. Since the amount of copper was a trace in the cases of XRD analysis, the copper nanoparticles peaks were not seen.

To determine the type of gas adsorption isotherm and pore volume of the prepared Zn-based compounds, their N_2_ adsorption–desorption isotherms were obtained at 77 K. According to Fig. 2C, a type II isotherm with an H3-type hysteresis ring was obtained through the N_2_ adsorption of the samples in the range *P/P*_o_ = 0.3–0.95, indicating their microporosity and plate-like shape of channels. Also, the total volume of cavities calculated at P/P_0_ = 0.990 was equal to 0.0036411 cm^3^g^−1^. Furthermore, Barrett-Joyner-Halenda's (BJH) analysis was conducted, and the average pore size of 1.8 nm was obtained for the prepared samples, which is in accordance with the pore size of microporous materials (Fig. 2C)^45,46^.

Figure 2D illustrates the UV–Vis spectroscopy of metal-free porphyrin (H_2_TPP) (Fig. 2Da), metal porphyrinato complex (ZnTPP) (Fig. 2Db), self-assembled ZnTPP (ZnTPP-NPs) (Fig. 2Dc), and nanocomposites (ZnTPP/Cu-NPs and ZnTPP/Cu-NPs-PAA) (Fig. 2Dd, De), in ethanol. The sharp Soret band at 420 nm and four Q bands at 516, 550, 592, and 650 nm were seen for the H_2_TTPP compound. Upon zinc coordinated to the porphyrin structure, the Soret band at 421.5 nm and two Q bands at 556 and 595 nm were observed with a red shift, indicating the molecular symmetry increased from D_2h_ to D_4h_, and the formation of ZnTPP complex was confirmed. The broadened Soret with two Q weak bands in the electronic absorption spectra of aggregated compounds (ZnTPP-NPs, ZnTPP/Cu-NPs, and ZnTPP/Cu-NPs-PAA) indicated the existence of metal–ligand coordination and π–π stacking interactions^41,42,47^.

**Figure 3:**
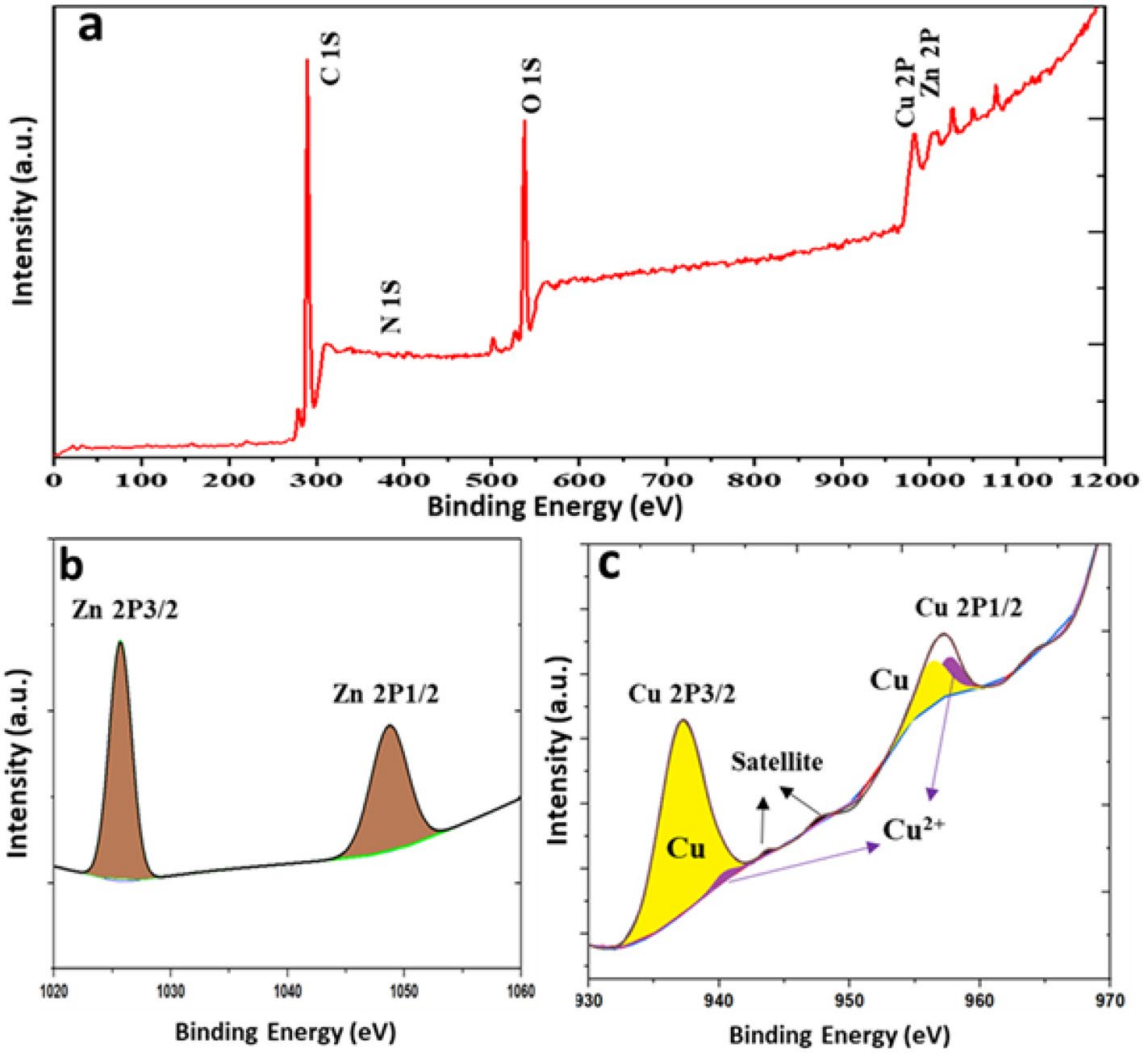
X-ray photoelectron spectroscopy survey analysis (**a**) and the high-resolution spectra of Zn 2p and Cu 2p (**b**), (**c**) respectively.

We utilized the freshly X-ray photoelectron spectroscopy (XPS) to survey the surface composition. The XPS analysis was the best tool to confirm the presence of Cu^0^ states in nanocomposites. Figure 3a displays the wide scan spectra of the ZnTPP/Cu-NPs (1), that was demonstrated the C 1s, N 1s, O 1s, Cu 2p, Zn 2p peaks. Figure 3b represents the high-resolution spectrum of Zn 2p, which is decomposed into two peaks at 1025.85 and 1048.58 eV, corresponding to Zn^2+^. The high-resolution spectrum of Cu 2p (Fig. 3c), displays two peaks of Cu 2p_3/2_ and Cu 2p_1/2_ which are decomposed into two peaks at 937.16 and 952.72 eV, corresponding to Cu^0^ with satellite peaks appeared at higher binding energy, indicating the existence of Cu^2+^. The ratio of Cu^0^ and Cu^2+^ has been calculated 95:5 respectively^48^.

**Figure 4:**
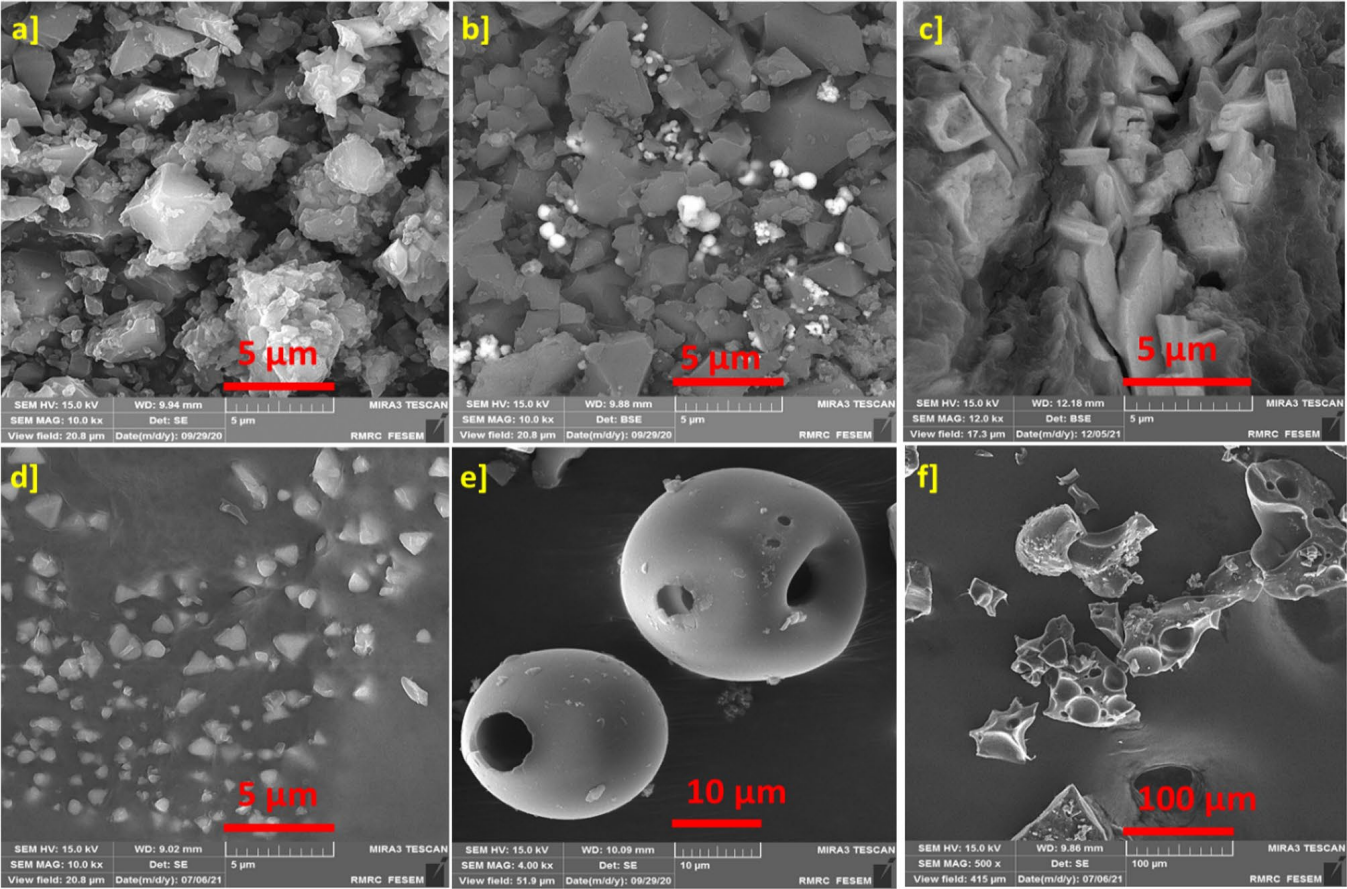
The FE-SEM images of various morpholines of synthesized samples: ZnTPP-NPs (**a**), ZnTPP /Cu-NPs (1) (**b**), ZnTPP /Cu-NPs (2) (**c**), ZnTPP/Cu-NPs (3) (**d**), ZnTPP/Cu-NPs-PAA-(1) (**e**) and ZnTPP/Cu-NPs-PAA (3) (**f**).

**Figure 5:**
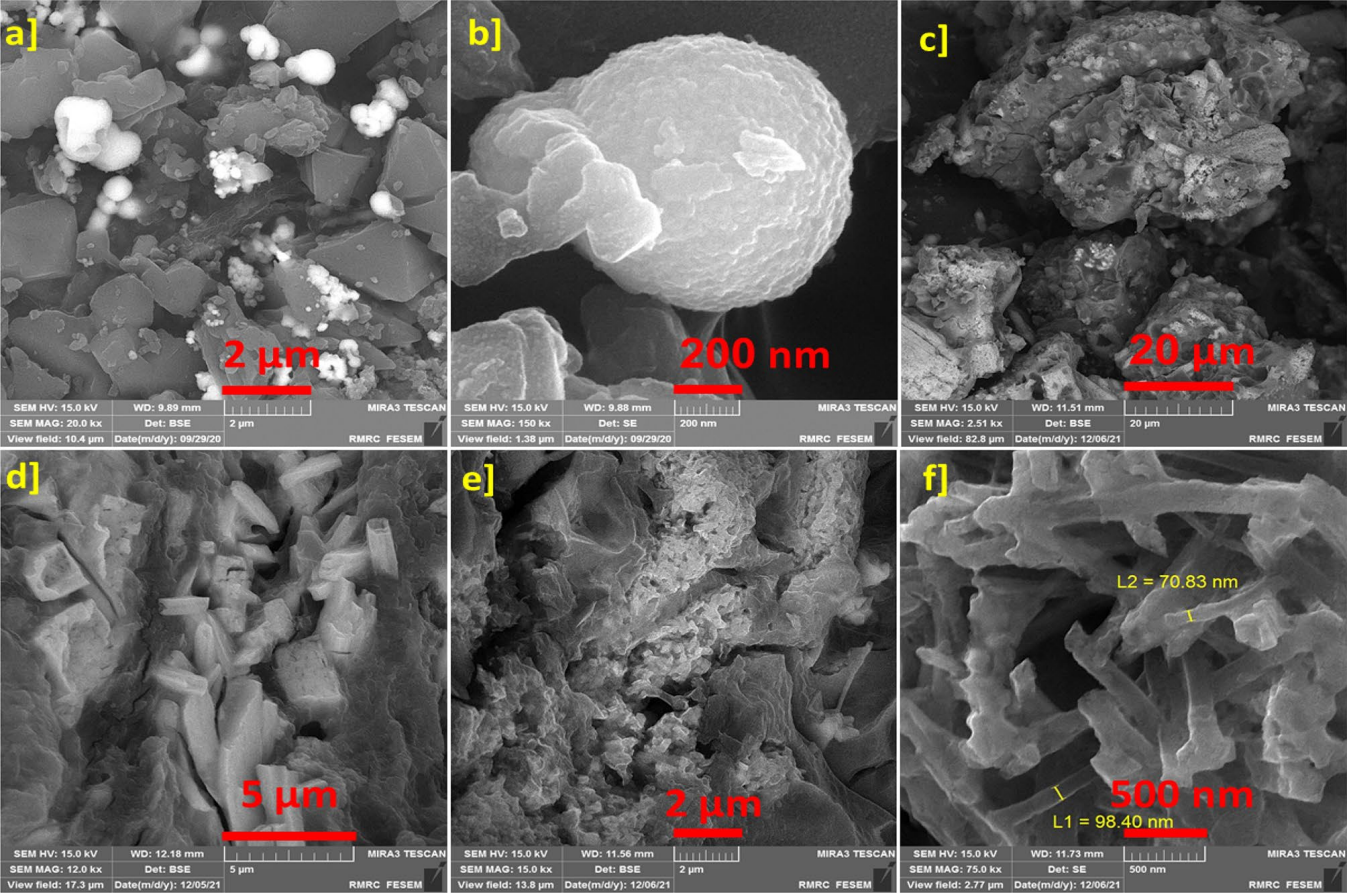
The FE-SEM images of ZnTPP/Cu-NPs (1) (**a**, **b**) and ZnTPP/Cu-NPs(2) (**c**–**f**), representing the growth of Cu nanoparticles.

**Figure 6:**
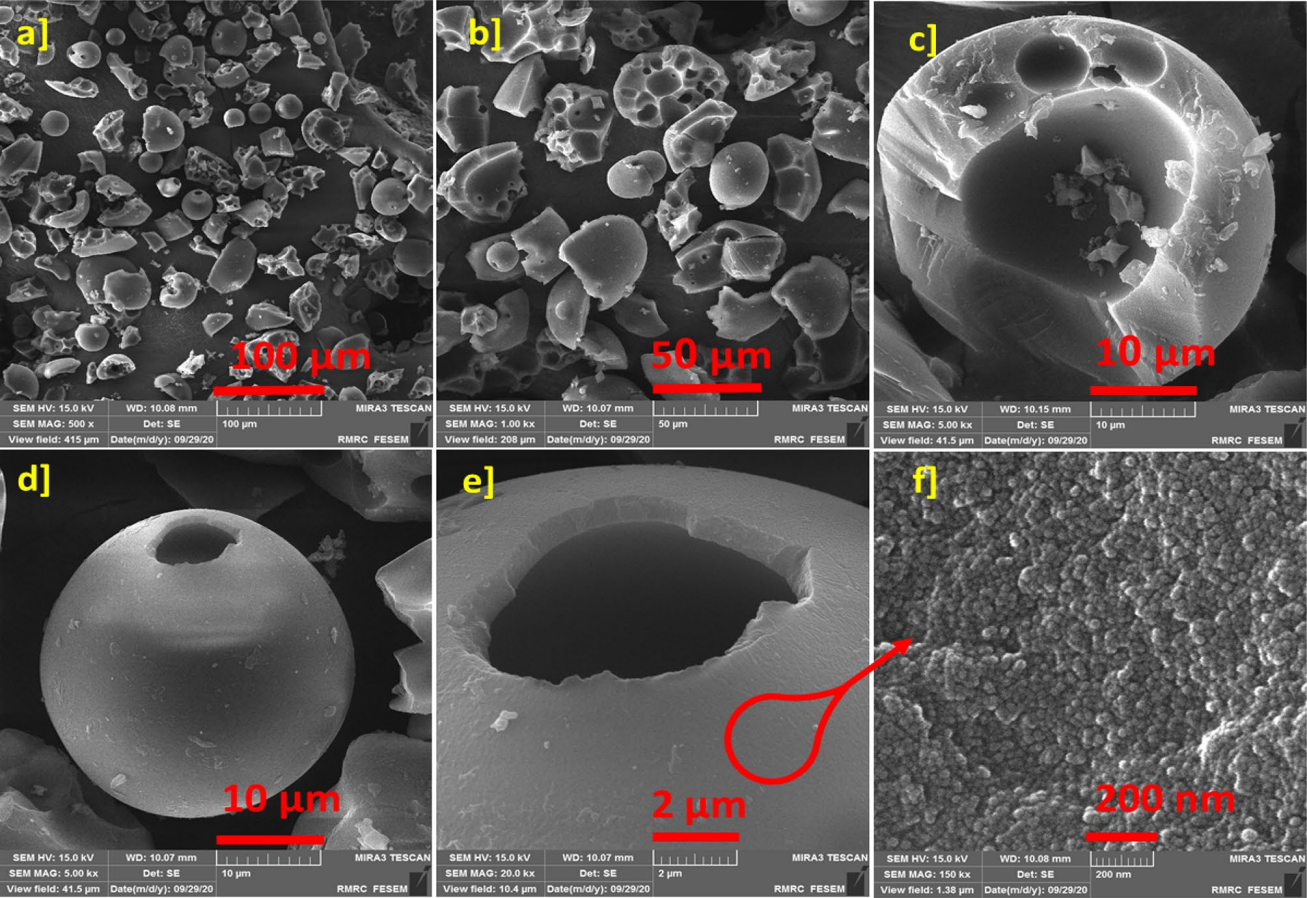
The FE-SEM images ZnTPP /Cu-NPs-PAA (1) (**a**–**f**) represent the porous spherical morphology from the route using PAA.

The synthesis of self-assembled ZnTPP (ZnTPP-NPs), ZnTPP-Cu-NPs, and ZnTPP/Cu-NPs-PAA composites was done under different conditions due to the ‏Table 1. Indicative FE-SEM images were achieved for the products with different conditions of synthesis. All of the morphologies formation and conversion of nanostructures during the synthesis of nanocomposites are shown in (Figs. 4, 5, 6).

Octahedral morphology was obtained for ZnTPP-NPs (Fig. 4a). ‏‏‏The synthesis of ZnTPP-NPs nanocomposites containing Cu NPs was accomplished with the present and absence of PAA as indicated in Table 1. The FE-SEM images of synthesized ZnTPP/Cu-NPs nanocomposite were shown in (Fig. 4b–d and Fig. 5a–f) which were not utilized PAA in the route of synthesis. The growth of sphere Cu nanoparticles on the porphyrin-based structure ascertains in the FE-SEM images of ZnTPP/Cu-NPs (1) compound (Fig. 5a, b). The continued growth of Cu nanoparticles formed the nanorods morphology in the case of ZnTPP/Cu-NPs (2) (Fig. 5d). Moreover, the porous spherical morphology was observed for ZnTPP/Cu-NPs-PAA (3) which was utilized Polyacrylic acid (PAA) in the synthesis as a modulator. (Fig. 6a–f)).

**Figure 7:**
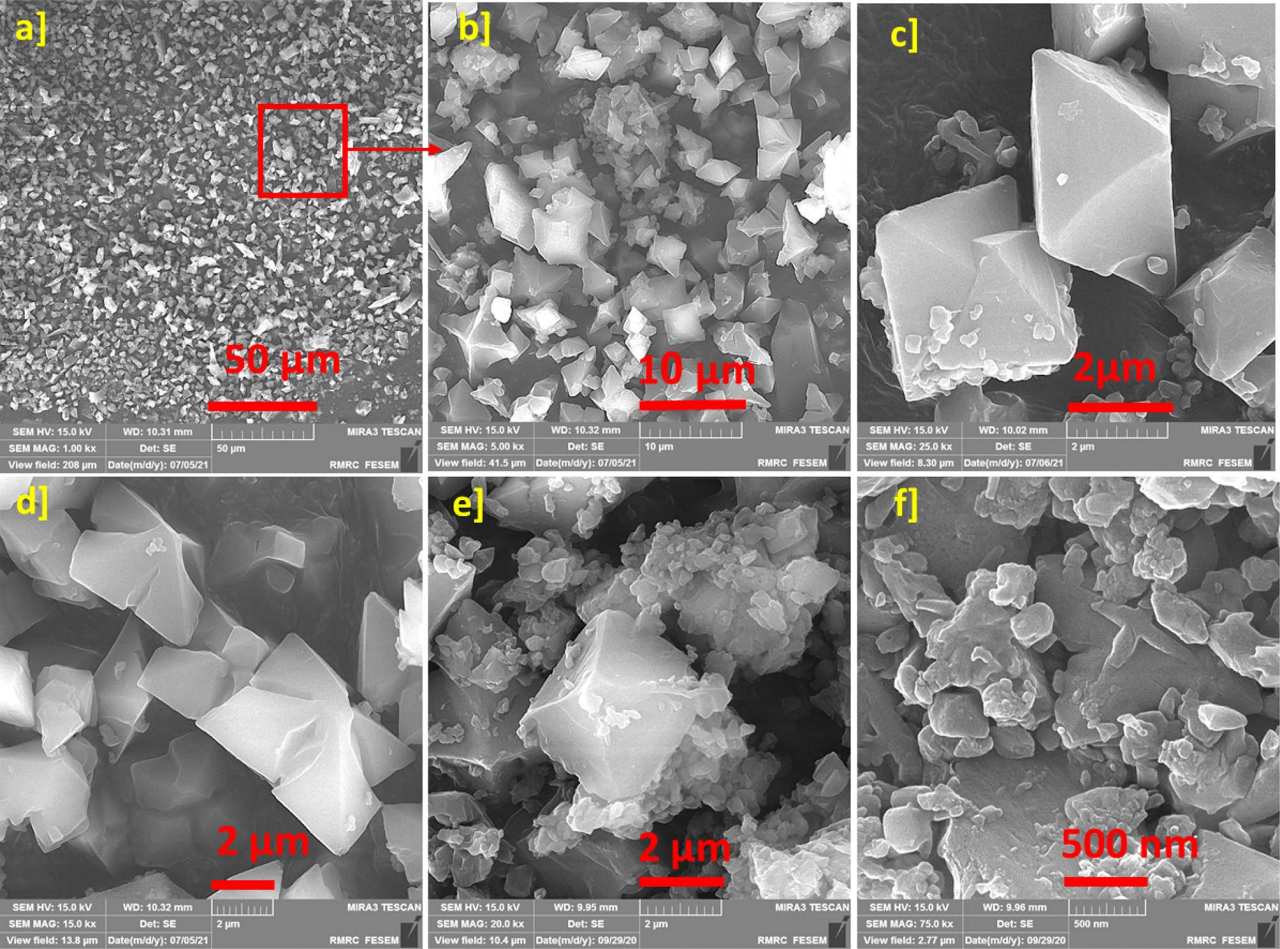
The FE-SEM image of ZnTPP-NPs after freeze-drying (**a**–**d**) and the FE-SEM images of ZnTPP-NPs before freeze-drying (**e**, **f**) for comparison.

It should be noted that the FE-SEM image of ZnTPP nanostructures was also recorded after freeze-drying. As (Fig. 7a–d) show, the nanostructures became more orderly and similar to accumulated octahedrons after freeze-drying compared with the structure of self-assembled ZnTPP (Fig. 7e, f).

**Figure 8:**
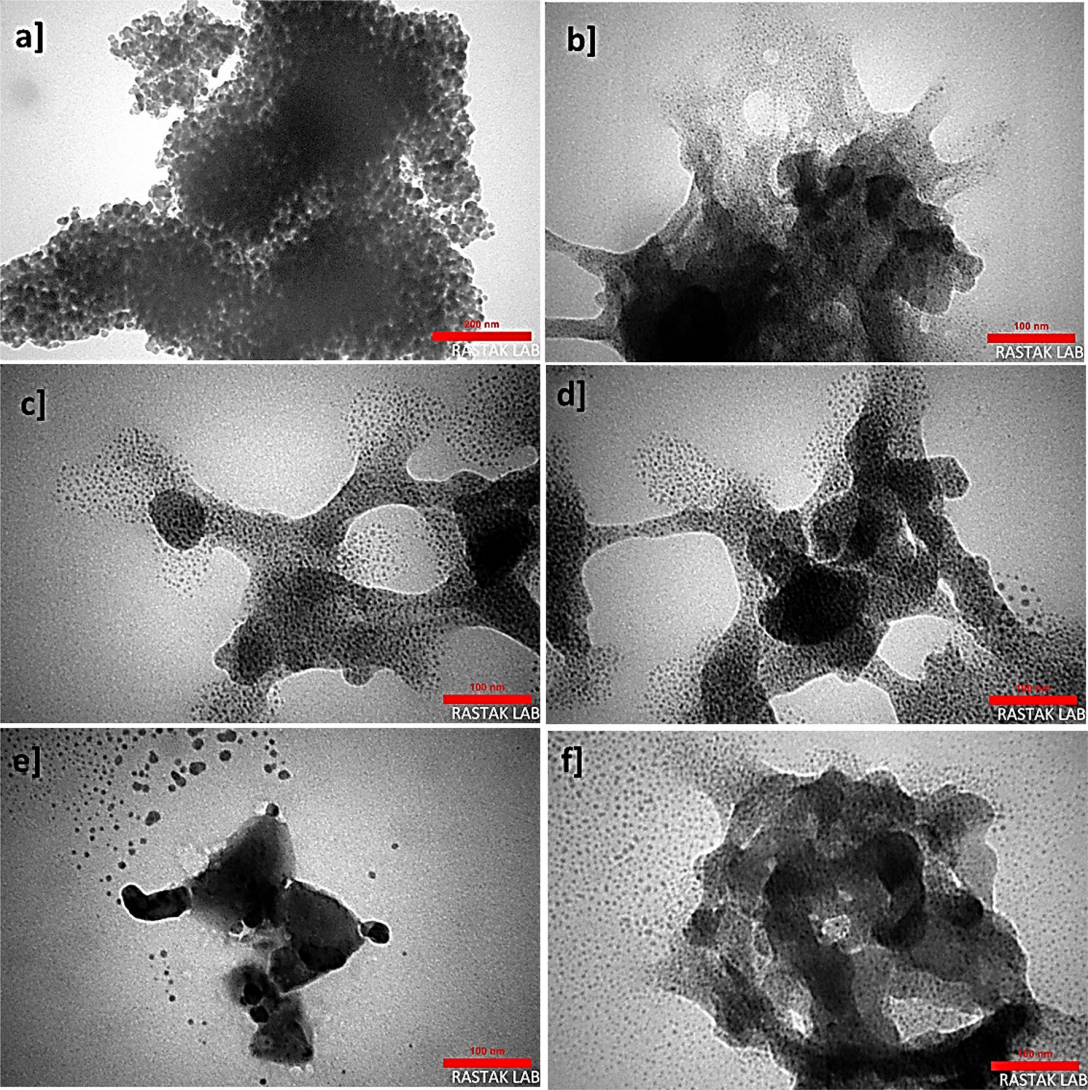
TEM images of ZnTPP/Cu-NPs (3) in the scale of 100 nm (**a**, **e**), ZnTPP/Cu-NPs(3) in the scale of 50 nm (**b**), ZnTPP/Cu-NPs-PAA(3) in the scale of 100 nm (**c**), and ZnTPP/Cu-NPs-PAA(3) in the scale of 200 nm (**d**).

The samples' transmission electron microscopy (TEM) images were also collected to investigate the size and morphology of the Cu nanoparticles in the nanocomposites body. The interconnected Cu-NPs with about 5 to 10 nm are observed within the nanocomposite structure (Fig. 8a–f)^9^.

**Figure 9:**
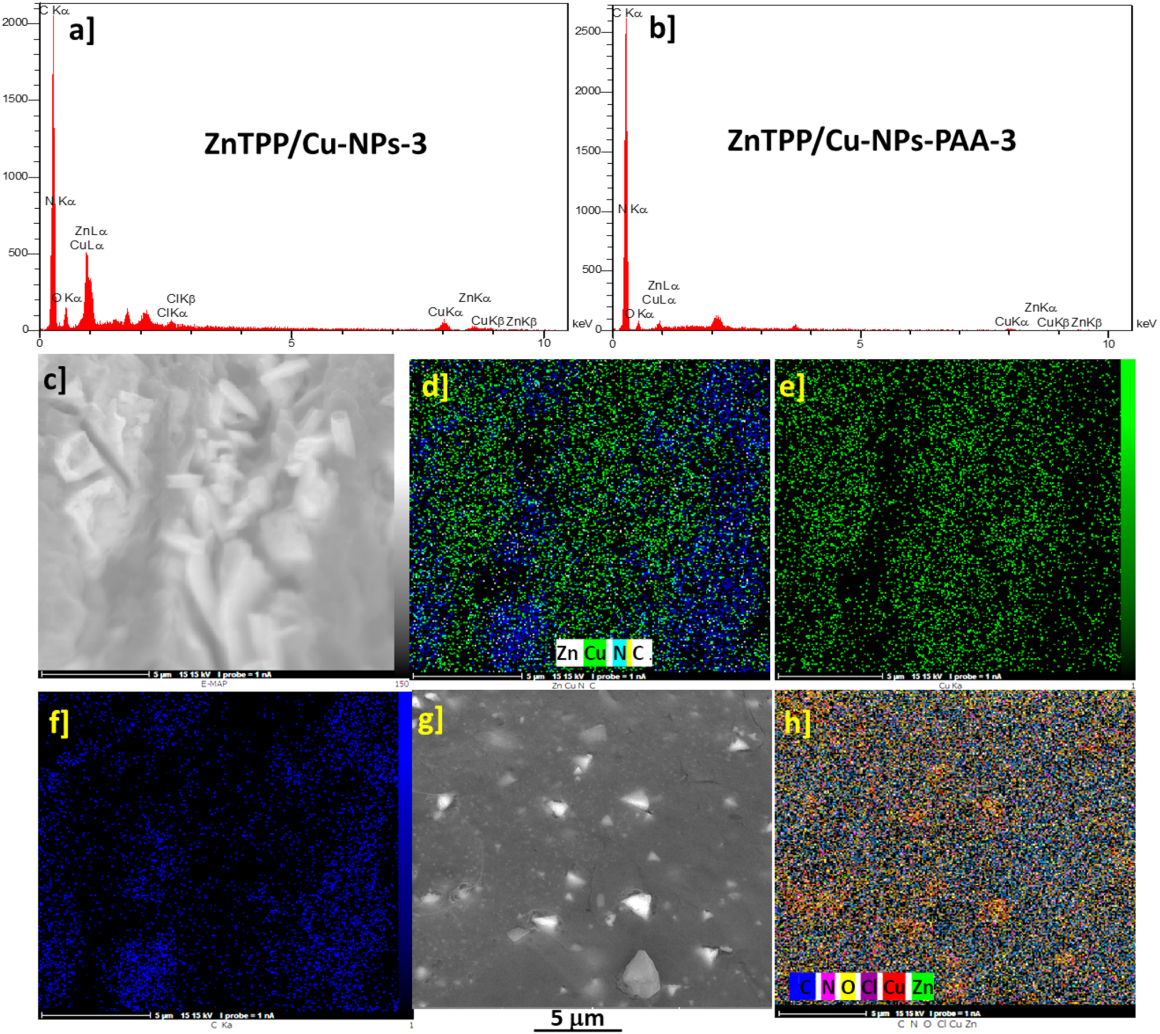
EDX analysis of ZnTPP/Cu-NPs (3) (**a**), EDX analysis ZnTPP/Cu-NPs-PAA (3) (**b**), elemental mapping spectra of ZnTPP/Cu-NPs(2) (**c**–**f**), and elemental mapping spectra of ZnTPP/Cu-NP-PAA-(3) (**g**, **h**).

Energy-dispersive x-ray spectroscopy (EDX) of the prepared ZnTPP/Cu-NPs showed both ZnTPP and Cu NPs components in it, demonstrating the successful synthesis of ZnTPP/Cu-NPs (Fig. 9a, b) Moreover the elemental mapping analysis are shown in (Fig. 9c–h) indicating the presence of CuNPs with a specified distribution in ZnTPP-Cu NPs and ZnTPP-Cu-NPs-PAA samples (Fig. 9c–h).

**Figure 10:**
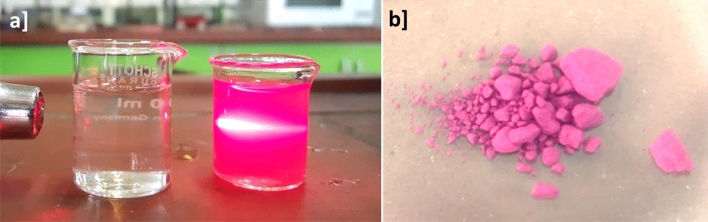
Light scattering experiment of colloidal ZnTPP/Cu-NPs (**a**) and prepared ZnTPP (**b**).

In order to determine the stability of suspended nanoparticles over time, a light scattering experiment was done. As (Fig. 10a) illustrates, firstly, each of the ZnTPP-NPs, ZnTPP/Cu-NPs-(1), and ZnTPP/Cu-NPs-PAA-(1) were dispersed in deionized water. Then, to compare, the prepared suspensions and a glass vessel of deionized water were exposed to the laser light. It was observed that the glass vessel containing the composite displayed the Tyndall effect, while the deionized water vessel showed no changes^49^. The synthesized ZnTPP was demonstrated in Fig. 10b as well. For colloid solutions DLS analysis is utilized to measure the nanoparticles size by the interaction between light and nanoparticles . The particle size distribution and zeta potential of synthesized ZnTPP/Cu-NPs (1) was represented in Fig. S2, the average sizes of ZnTPP/Cu-NPs (1) were 264.3 nm. Generally The obtained size of nanoparticles from DLS analyses bigger than other analyses such as TEM and XRD.This phenomena is attributed to the effected of metallic core, coating, and stabilizer substances which stack on the surface of nanoparticles^50^.

### Antibacterial tests

**Figure 11:**
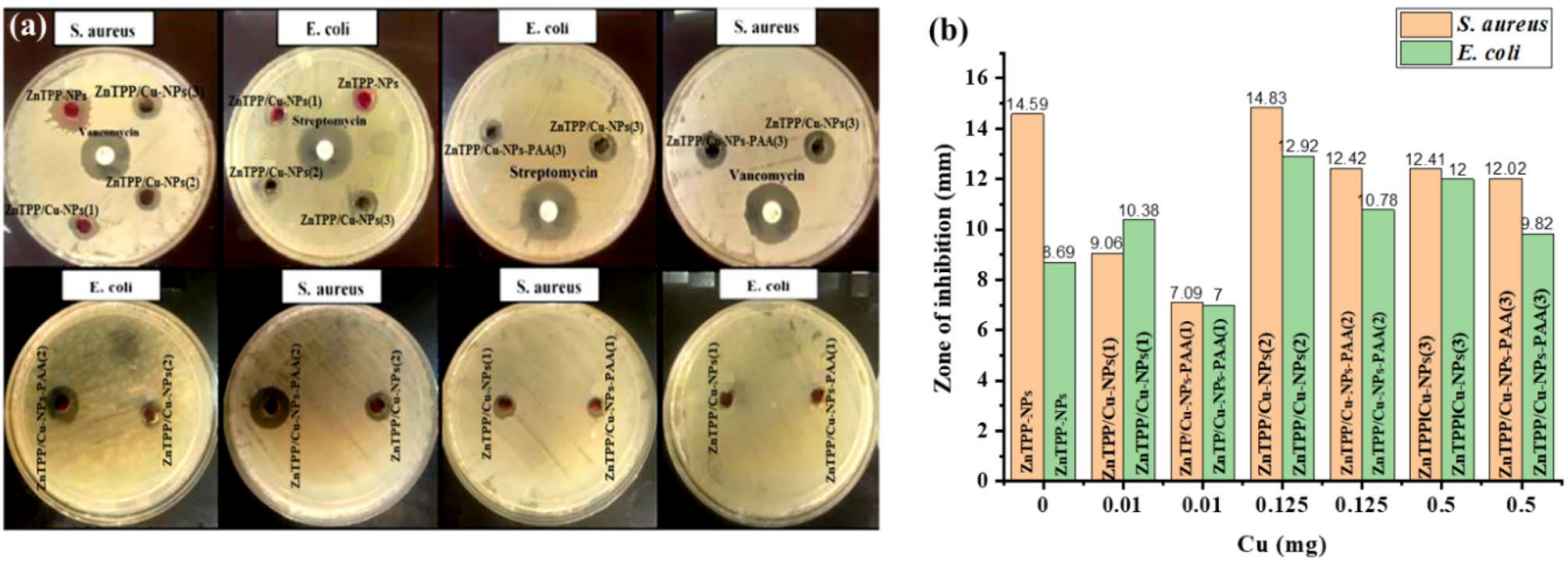
The well diffusion disks for all samples against *E. coli* (ATCC 25922) and *S. aureus* (ATCC 25923) bacteria (**a**). Bar graph for the average zones of inhibition (**b**).

This study was carried out to screen the antibacterial properties of synthesized samples against gram-negative *E. c oli* (ATCC 25922) and gram-positive *S. aureus* (ATCC 25923) bacteria using the agar well diffusion method (Fig. 11a).

The obtained results showed the significant role of nanocomposites on the growth inhibition of *E. coli* (ATCC 25922) and *S. aureus* (ATCC 25923) bacteria. The average zones of inhibition of growth diameter were obtained three times of repeat, and the results were illustrated in Table 2. Furthermore, the bar graph of the zone of inhibition of growth diameter was represented in (Fig. 11b and Fig. S2). The largest obtained zone of inhibition of diameter of *S. aureus* (ATCC 25923) are attributed to the ZnTPP/Cu-NPs (2) with nanorod morphology, (14.83 mm) and ZnTPP-NPs with octahedron morphology, (14.59 mm) respectively. Moreover, the zone of inhibition of diameter obtained for *E. coli* (ATCC 25922) bacteria treated with nanorod ZnTPP/Cu-NPs (2), was 12.92 mm. The results showed the reduction of the zone of inhibition of diameter for both *E. coli* (ATCC 25922) and *S. aureus* (ATCC 25923) bacteria treated with the nanocomposites containing PAA. In addition, the results demonstrated that the ZnTPP-NPs and all synthesized nanocomposites have more efficient antibacterial performance for *S. aureus* (ATCC 25923). In previous studies has been shown that most of the porphyrin molecules efficiently bind to Gram-(+) bacteria and inactivate them. but the Gram-(-) bacteria are seemed to be more resistant in treating with porphyrin .Since a porous layer of peptidoglycan and lipoteichoic acid in the Gram-positive bacteria cell wall is present, allowing to diffuse better for porphyrin molecules occurred^50–52^.

**Table 2:** Average zone of inhibition of samples.

Samples	Average zone of inhibition *S. aureus* (ATCC 2593) (mm)	Average zone of inhibition *E. coli* (ATCC 25922) (mm)
ZnTPP-NPs	14.59	8.69
ZnTPP/Cu-NPs (1)	9.06	10.38
ZnTPP/Cu-NPs-PAA (1)	7.09	7.00
ZnTPP/Cu-NPs (2)	14.83	12.92
ZnTPP/Cu-NPs-PAA (2)	12.42	10.78
ZnTPP/Cu-NPs (3)	12.41	12.00
ZnTPP/Cu-NPs-PAA (3)	12.02	9.82

MIC and MBC test results were conducted to investigate the antibacterial properties of the prepared samples. In this way, both ZnTPP-NPs and ZnTPP/Cu-NPs-PAA with concentrations of 0.0390, 0.07812, 0.15635, 0.3125, 0.625, 1.25, 2.5, and 5 mg/mL were separately added to the corresponding wells in a 96-well plate (Fig. S3). The MIC and MBC values for ZnTPP-NPs against *E. coli* (ATCC 25922) and *S. aureus* (ATCC 25923) were obtained at 0.625 mg/mL. Porphyrins have lots of capability to improve as antibacterial and anti-cancer drugs. Obtained octahedron nanoparticles of ZnTPP improved to apply as therapeutic drugs. Since the obtained MIC and MBC for synthesized ZnTPP-NPs for *S. aureus* (ATCC 25923) and *E. coli* (ATCC 25922) were as the same, the wide utilization of ZnTPP-NPs as antibiotics with an inhibition mechanism simultaneous with killing bacteria, is proposed.

**Figure 12:**
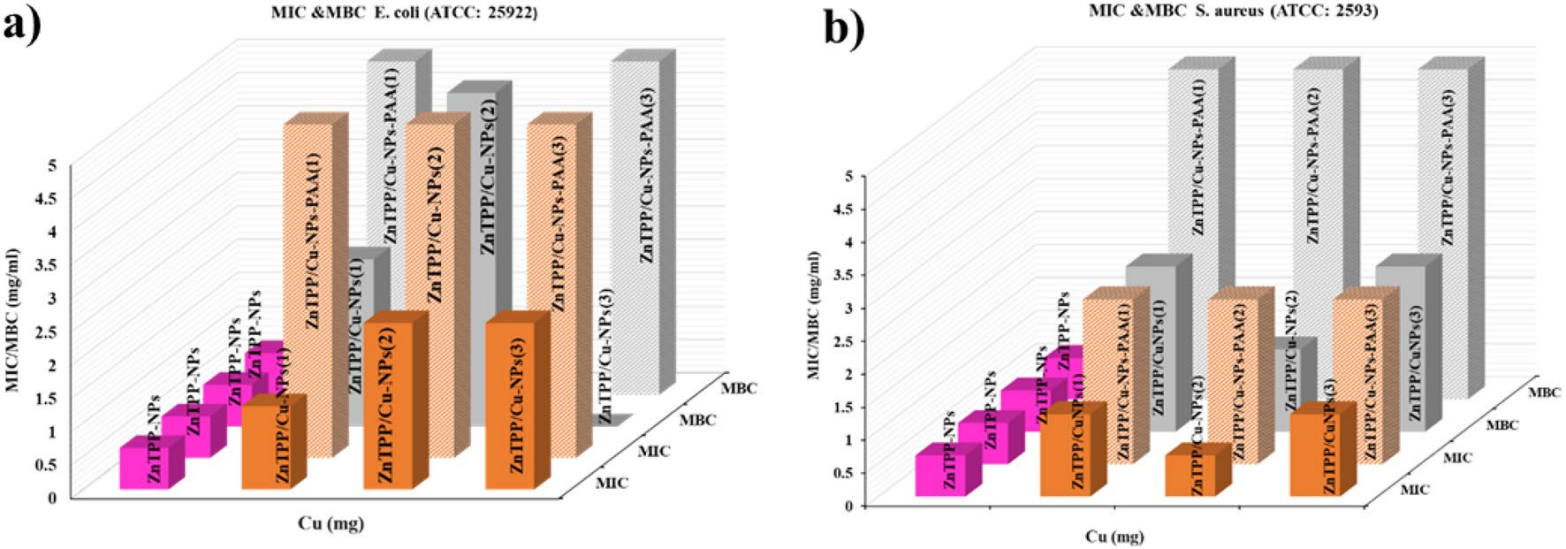
Column chart representing MIC and MBC values of *S. aureus* (ATCC 25923) and *E. coli* (ATCC 25922).

The MIC and MBC results for all synthesized ZnTPP/Cu nanocomposites are illustrated in Fig. 12. The MIC and MBC values of the synthesized samples with the different amounts of Cu have been displayed in Table 3. Nanomaterials with edges such as nanorods have favorable antibacterial functions^53^. The ZnTPP/Cu-NPs (2) with nanorod had the least amount of MIC value for *S. aureus* (ATCC 25923) between the synthesized nanocomposites. The results of MIC and MBC tests demonstrated the non-dependency of the MIC value on the amount of utilized Copper for PAA modulated nanocomposites for both *S. aureus* (ATCC 25923) and *E. coli* (ATCC 25922) bacteria. These results were in accordance with the zone of inhibition of diameter results.

**Table 3:** MIC and MBC value for *E. coli* and *S. aureus* strains for all samples.

Composite	*E. coli* (ATCC 25922)		*S. aureus* (ATCC 2593)		*E. coli* (PTCC 1399)		*S. aureus* (PTCC 1112)	
	MIC^a^	MBC ^a^	MIC	MBC	MIC	MBC	MIC	MBC
ZnTPP-NPs	0.625	0.625	0.625	0.625	2.5	2.5	2.5	–
ZnTPP/Cu-NPs (1)	1.25	2.5	1.25	2.5	–	–	–	–
ZnTPP/Cu-NPs (2)	2.5	5	0.625	1.25	–	–	–	–
ZnTPP/Cu-NPs (3)	2.5	–^b^	1.25	5	1.25	1.25	2.5	–
ZnTPP/Cu-NPs-PAA (1)	5	5	2.5	5	–	–	–	–
ZnTPP/Cu-NPs-PAA (2)	5	–	2.5	5	–	–	–	–
ZnTPP/Cu-NPs-PAA (3)	5	5	2.5	5	2.5	2.5	1.25	2.5

^a^For all MIC and MBC the concentration is mg/mL.

^b^In the table the (−) means “Not done”.

**Figure 13:**
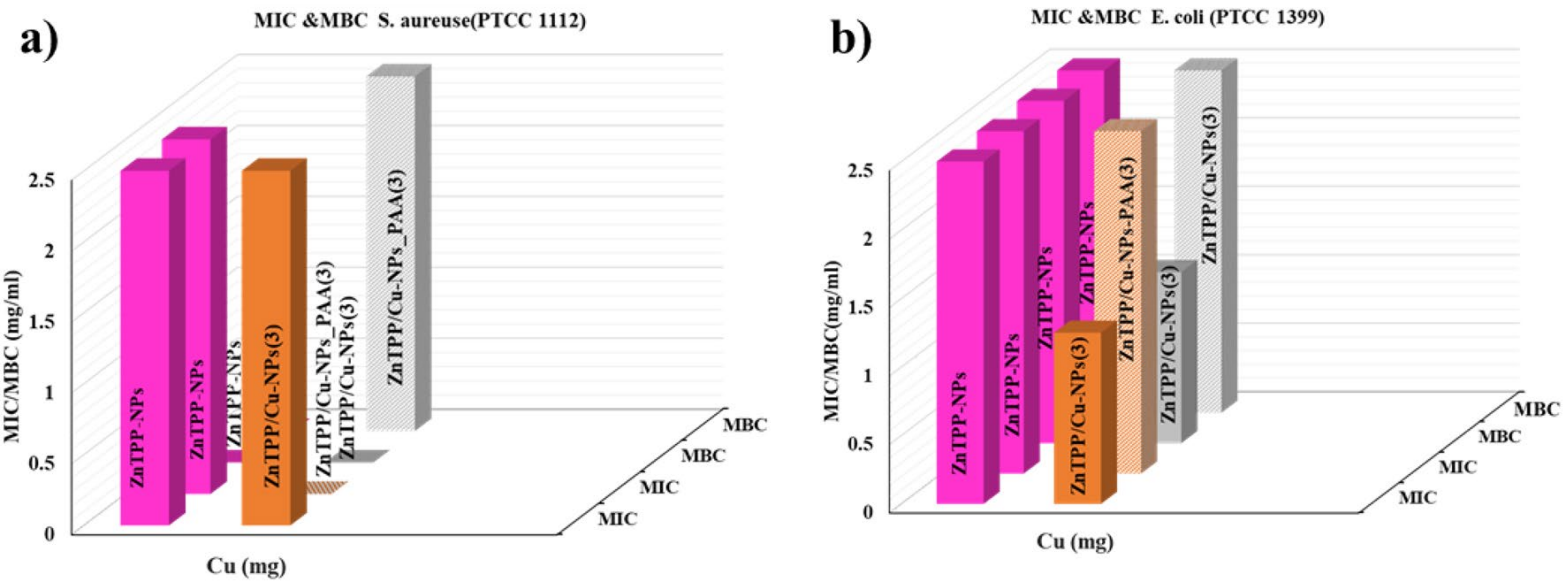
Column chart representing MIC and MBC values of *S. aureus* (PTCC1112) and *E. coli* (PTCC1399).

Three kinds of synthetic compounds (ZnTPP-NPs, ZnTPP/Cu-NPs (3), and ZnTPP/Cu-NPs-PAA (3)) were selected, and their MIC and MBC values were obtained for *E. coli* (PTCC 1399) and *S. aureus* (PTCC 1112) strains (Fig. 13) to assess the strain performance of products. The results showed that the ZnTPP-NPs, ZnTPP/Cu-NPs (3), and ZnTPP/Cu-NPs-PAA (3) are the strain dependent antibacterial agents.

The general pathways activities for the Cu NPs antimicrobial activity are: (1) interference during cell wall synthesis; (2) suppression during protein biosynthesis (translation); (3) interference or disruption of transcription process; and (4) disruption of primary metabolic pathways. The size of the nanoparticles also play a vital role in antimicrobial property as the cell wall is more bare to nanoparticles through outer membrane indeed death of bacterium occurs because of high volume ratio of copper nanoparticles enables to interact with the bacterial cell membrane through its surface^54^. The metal complexation increases the porphyrin chemical stability and may enhance their interaction with cell membranes^55^. The antibacterial investigation of different synthesized morphologies of ZnTPP/Cu-NPs indicated that the MIC and MBC values were dependent on their morphology.

### Cytotoxicity assay results

**Figure 14:**
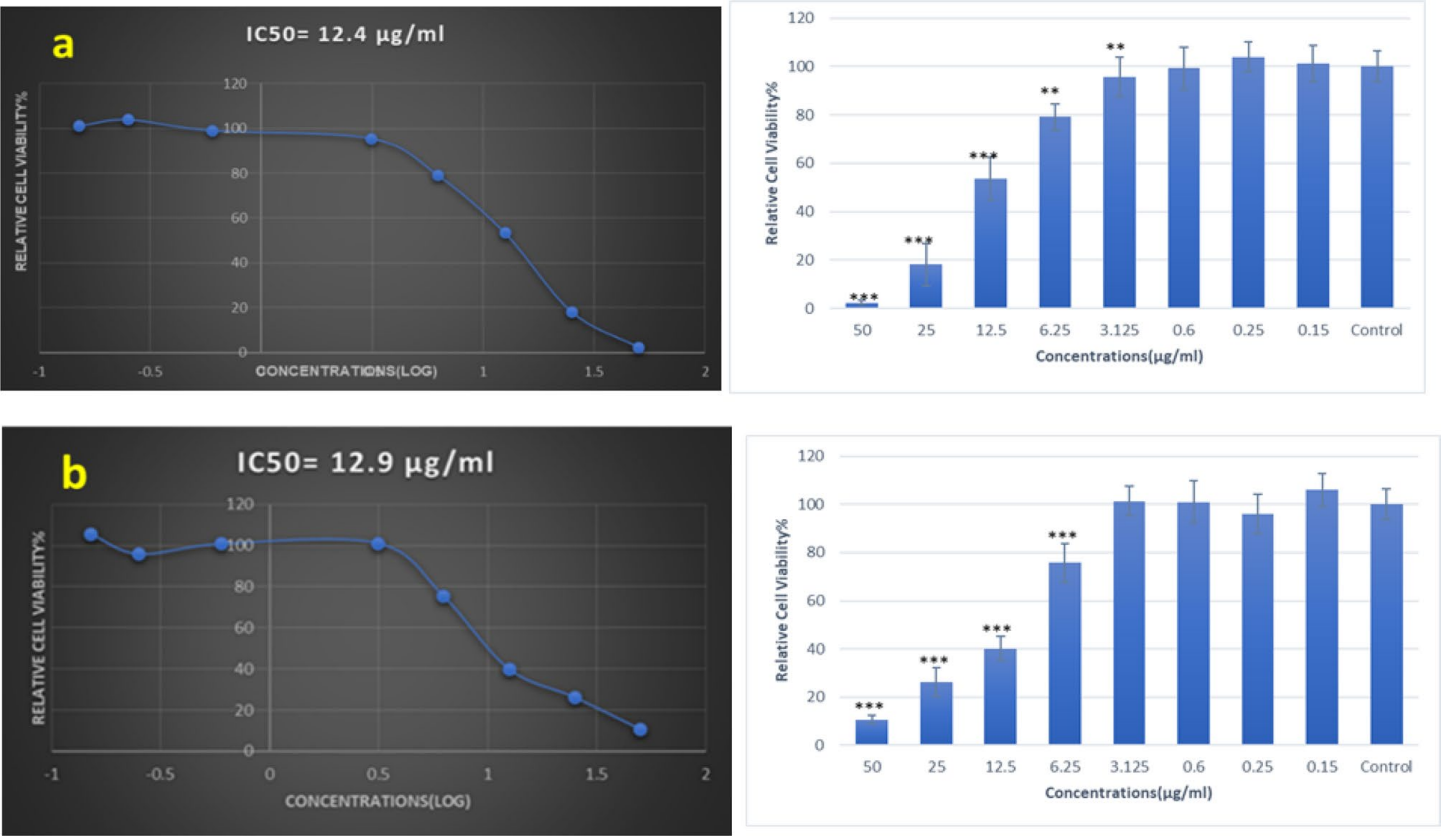
The viability of MCF-7 cancer cells, 24 h after treatment with ZnTPP/Cu-NPs (3) (**a**) and ZnTPP/Cu-NPs-PAA (3) (**b**).

**Figure 15:**
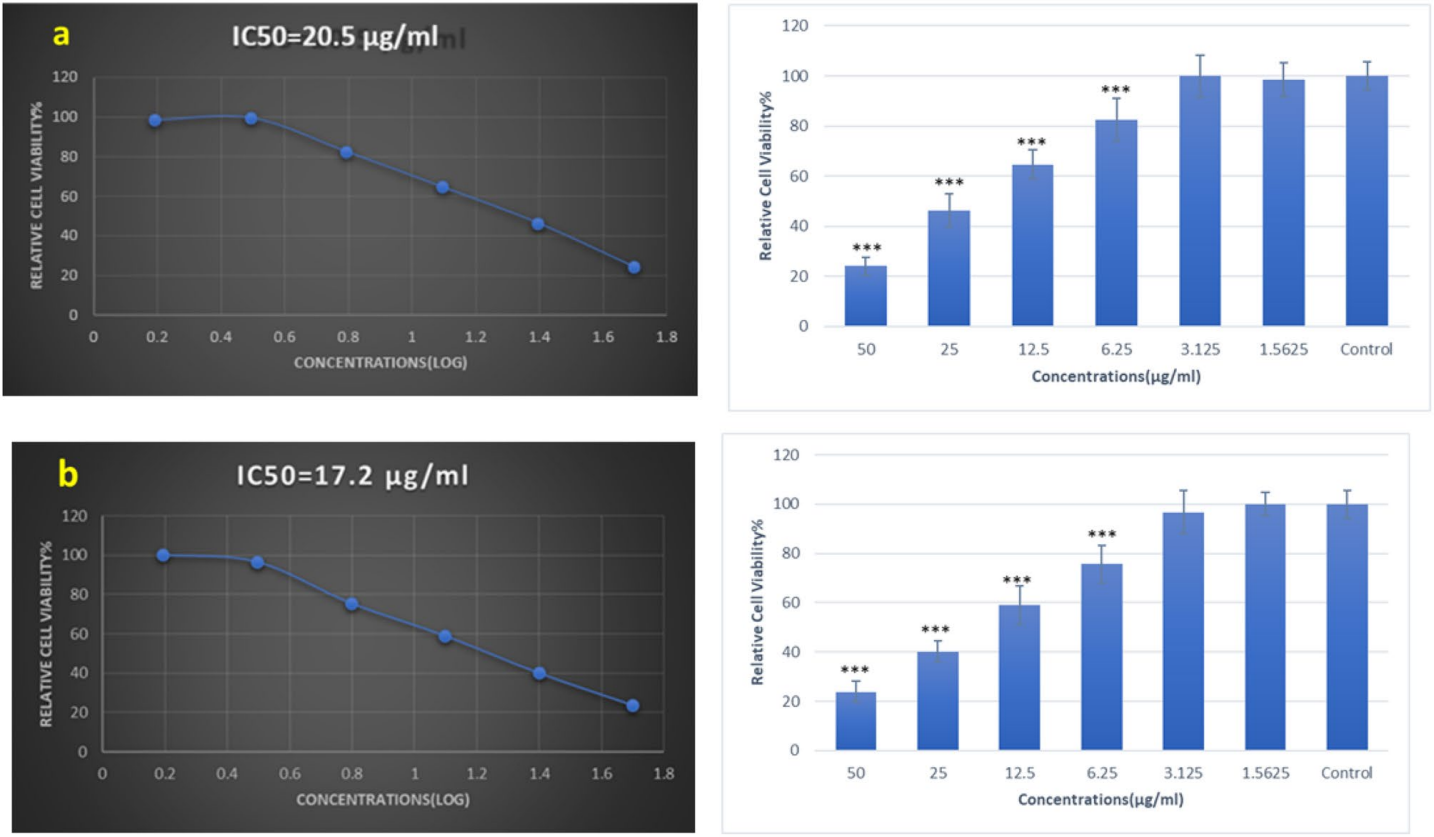
The viability of HEK-293 cells, 24 h after treatment with ZnTPP/Cu-NPs (3) (**a**) and ZnTPP/Cu-NPs-PAA (3) (**b**).

The cytotoxicity of some nanomaterials like Cu and CuO nanoparticles are dose-dependent^56^. The inhibition of MCF‐7 and HEK-293 cells after 24 h of incubation with the concentrations of ZnTPP/CuNPs (3) and ZnTPP/Cu-NPs-PAA (3) was dependent on doses. The cytotoxicity The half-maximal inhibitory concentration (IC50) values of ZnTPP/Cu-NPs (3) and ZnTPP/Cu-NPs-PAA (3) were 12.4 and 12.9 μg/mL for MCF‐7 cells in 24 h respectively. However, the obtained IC50 values on HEK-293 cells treated with ZnTPP/Cu-NPs (3) and ZnTPP/Cu-NPs-PAA (3) are 20.5 and 17.2 μg/mL in 24 h of incubation, respectively (Fig. 14a, b) and (Fig. 15a, b). The images of the 96-well plate of MTT assay treated with ZnTPP/Cu-NPs (3) and ZnTPP/Cu-NPs-PAA (3) on MCF-7 cells and HEK-293 cells are in Fig. S4a,b. In the present study, the cell viability assay indicated that ZnTPP/Cu-NPs were severely toxic to human breast cancer cells. In order to compare the performance of nanocomposites on normal cells, the HEK-293 epithelial cells were used. The MTT test demonstrated the IC50 value of ZnTPP/Cu-NPs (3) on HEK-293 cells is more than on cancer cells.

## Conclusion

In summary, this study introduces a green and simple method using ZnTPP as a precursor with photochemical functionality to synthesize ZnTPP/Cu-NPs with antibacterial activity and breast cancer cytotoxic properties. In this regard, firstly colloidal ZnTPP-NPs were synthesized and utilized as a promoter to synthesize ZnTPP/Cu-NPs. Utilizing PAA as a modulator in nanocomposite syntheses led to reduced antibacterial activity. Antibacterial activity of colloidal ZnTPP-NPs and nanocomposites was investigated by determining the zone of inhibition of diameter, MIC, and MBC values against different strains of *E. coli* and *S. aureus* bacteria. The MIC and MBC values of octahedron ZnTPP-NPs for both *E. coli* (ATCC 25922) and *S. aureus* (ATCC 25923) bacteria were achieved at the same concentration. The results showed the diverse antibacterial performance of different morphologies of synthesized ZnTPP/Cu-NPs. The best antibacterial performance of nanocomposites is attributed to the ZnTPP/Cu-NPs with nanorod morphology. Moreover, ZnTPP/Cu-NPs have a severe cytotoxic effect against breast cancer cells with an IC50 value less than the obtained IC50 for HEK-293 cells. As a result, the synthesized nanocomposites with the mentioned properties are capable to improve and utilize in antibacterial photodynamic therapy and breast cancer treatment.

## Supplementary Information

Supplementary Information.

## Data Availability

All data generated or analyzed during this study are included in this published article (and its Supplementary Information files).
