# Supplementary material for: Zn (II)-porphyrin-based photochemically green synthesis of novel ZnTPP/Cu nanocomposites with antibacterial activities and cytotoxic features against breast cancer cells
Source: Sci Rep. 2022 Oct 12;12:17121. doi: 10.1038/s41598-022-21446-3 (PMC9556751; doi:10.1038/s41598-022-21446-3)
Supplement: Supplementary file 1 — Supplementary Information. [file 41598_2022_21446_MOESM1_ESM.docx]

**Electronic Supporting Information**

**Zn(II)-porphyrin-based photochemically green synthesis of novel ZnTPP/Cu nanocomposites with antibacterial activities and cytotoxic features against breast cancer cells**

Sajedeh Tehrani Nejad, ^a^ Rahmatollah Rahimi, *^,a^ Mahboubeh Rabbani, ^a^ Sadegh Rostamnia *^,b^

*^a^ Department of Chemistry, Iran University of Science and Technology, 16846-13114, Tehran, Iran. Email:* [*rahimi_rah@iust.ac.ir*](mailto:Rahimi_rah@iust.ac.ir)

*^b^ Organic and Nano Group (ONG), Department of Chemistry, Iran University of Science and Technology (IUST), PO Box 16846-13114, Tehran, Iran. Email: rostamnia@iust.ac.ir*

**Overall photodegradation results**

Fig. S1 demonstrates that the MB solution was photodegraded (55.86%) with ZnTPP/Cu Nps (A_1_) as well as (85.80%) with ZnTPP/Cu NPs (A_2_).


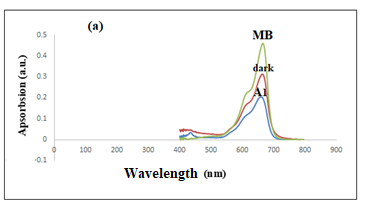

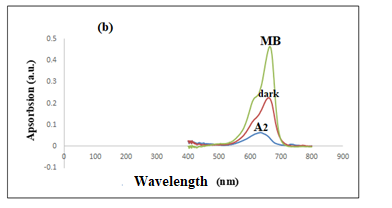


**Fig. S1.** Photodegradation of MB (a) ZnTPP/Cu NPs composite (A_1_) is photocatalyst and (b) ZnTPP/Cu NPs composite (A_2_) is photocatalyst.


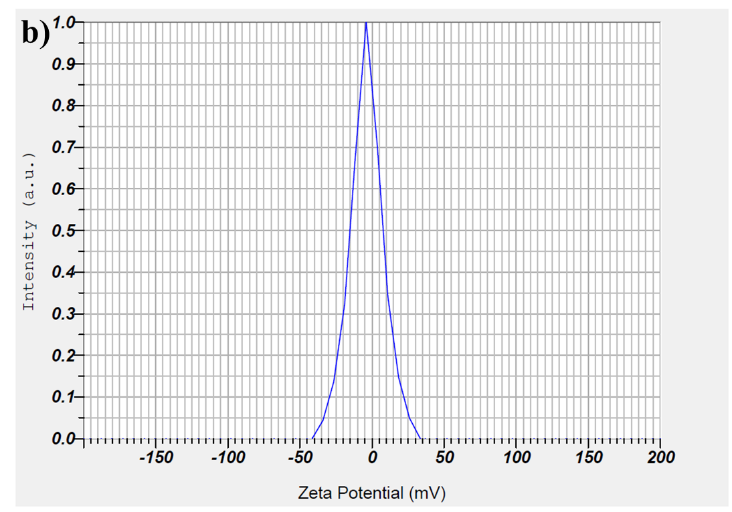

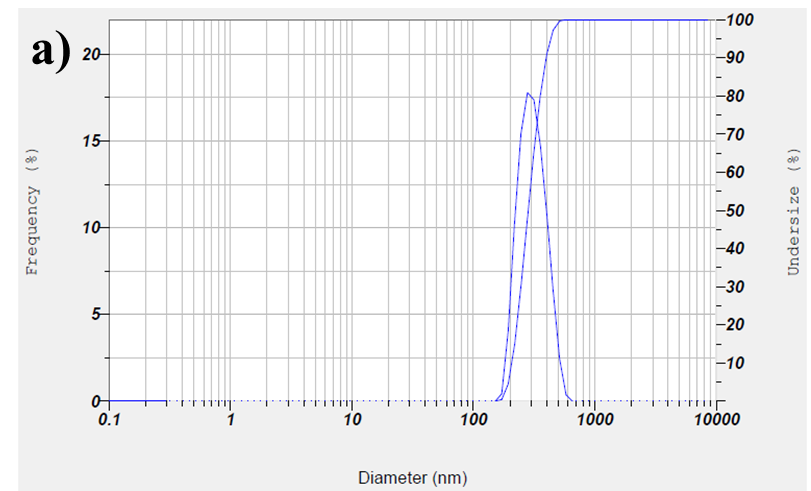


**Fig. S2.** DLS analysis: particle size distribution (a) and zeta potential of synthesized ZnTPP/Cu-NPs (2)

**Fig. S3.** Bar graph for the average zones of inhibition.


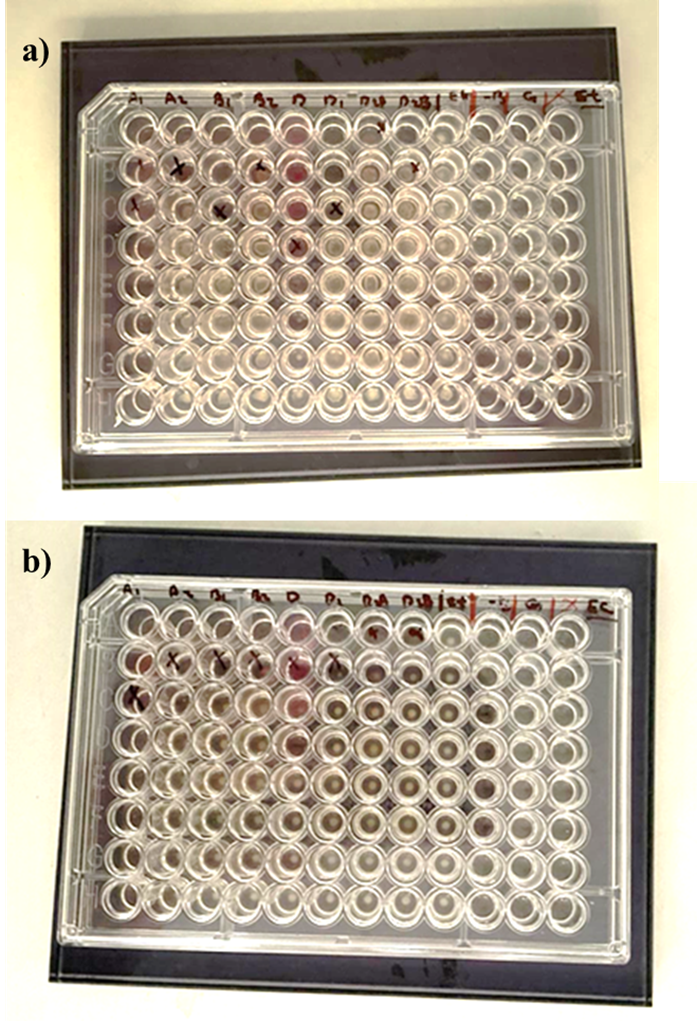


**Fig. S4.** The micro plates that representative the MIC test (a) for S. aureus and (b) for E. coli.


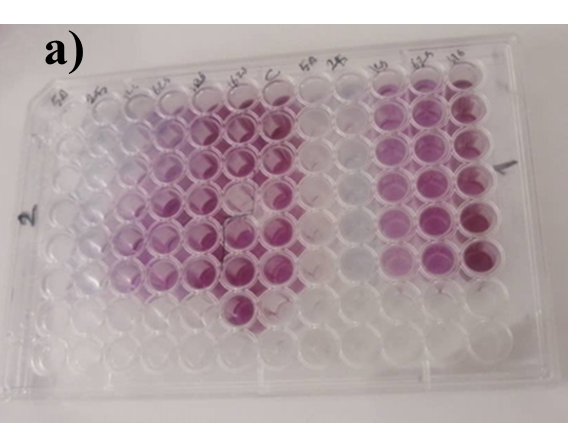

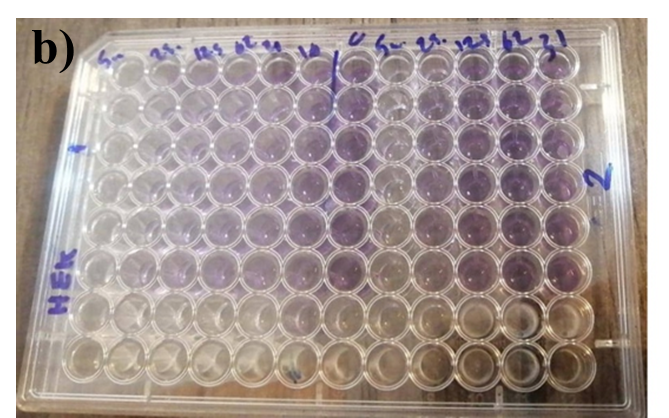


**Fig. S5.** The images of 96-well plate of MTT assay treated with ZnTPP/Cu-NPs and ZnTPP/Cu-NPs-PAA on MCF-7 cells (a) and HEK-293 cells (b)
